# Multiomic Profiling Reveals the Regulation of Many Immune-Related Genes by PU.1 in Porcine Alveolar Macrophages

**DOI:** 10.3390/ani16071116

**Published:** 2026-04-05

**Authors:** Jiayao Jiang, Juan Yang, Liangliang Zhang, Yanhua Li, Chenyang Tang, Chenxi Li, Yuzhuo Li, Hairui Fan, Cui Du, Mingan Sun

**Affiliations:** 1 Institute of Comparative Medicine, College of Veterinary Medicine, Yangzhou University, Yangzhou 225009, China; dx120220184@stu.yzu.edu.cn (J.J.); mx120231037@stu.yzu.edu.cn (J.Y.); zll05101534@163.com (L.Z.); 007206@yzu.edu.cn (Y.L.); 13937816217@163.com (C.T.); 007659@yzu.edu.cn (C.L.); mx120210971@stu.yzu.edu.cn (Y.L.); hairuifan@yzu.edu.cn (H.F.); 2 Joint International Research Laboratory of Important Animal Infectious Diseases and Zoonoses of Jiangsu Higher Education Institutions, Yangzhou 225009, China; 3 Jiangsu Co-Innovation Center for Prevention and Control of Important Animal Infectious Diseases and Zoonosis, Joint International Research Laboratory of Agriculture and Agri-Product Safety of Ministry of Education of China, Yangzhou 225009, China; 4 Interdisciplinary Center for Zoonotic Diseases and Biosafety, Yangzhou University, Yangzhou 225009, China

**Keywords:** alveolar macrophage, pig, PU.1, immune, transcription factor, enhancer, transposable element, CUT&Tag, RNA-seq

## Abstract

Alveolar macrophages (AMs) play essential roles in maintaining homeostasis and immunity in the lung. The transcription factor PU.1, encoded by *SPI1*, is a core regulator in multiple immune cell lineages. However, its binding property and regulatory role in AMs remain unclear. The pig serves as an important livestock species and a valuable biomedical model. Using porcine AMs (PAMs) as a model, we combined gene knockdown experiments with multiomic profiling to elucidate the regulatory role of PU.1 in AMs. By integrating the RNA-seq data before and after *SPI1* knockdown, we demonstrate that disruption of PU.1 impairs the expression of numerous immune-related genes, including many crucial for innate immune responses. We further employed CUT&Tag to characterize the genome-wide occupancy of PU.1 and the active histone modification H3K27ac, and found that PU.1 primarily binds active cis-regulatory elements (CREs), including a large proportion of enhancers derived from transposable elements. Moreover, integrative analysis identifies a set of CREs and their associated genes, which are putative direct targets of PU.1. Overall, this study provides novel insights into the regulatory role of PU.1 in AMs and extends our knowledge about this core regulator in the mammalian immune system.

## Simple Summary

The pig serves as an important livestock species and a valuable biomedical model. Alveolar macrophages (AMs) are essential for maintaining homeostasis and immunity in the lung. Using porcine alveolar macrophages as a model, this study elucidates the regulatory role of the core myeloid transcription factor PU.1. By integrating *SPI1* knockdown with multiomic profiling, we systematically mapped the genome-wide binding landscape and regulatory network governed by PU.1. We demonstrate that PU.1 predominantly binds active cis-regulatory elements, including many enhancers derived from transposable elements. Importantly, disruption of PU.1 causes disrupted expression of numerous immune-related genes—including many likely under the direct regulation of PU.1, thereby indicating the potential role of PU.1 in regulating AM immunity. Our findings provide novel insights into the regulatory role of PU.1 in AMs and expand the understanding of this master regulator in the mammalian immune system.

## 1. Introduction

Alveolar macrophages (AMs), the most abundant innate immune cells residing in the alveolar spaces, play crucial roles in maintaining lung homeostasis and orchestrating innate immunity [1,2,3]. As the respiratory tract is constantly exposed to environmental insults, AMs serve as the primary defense against a wide array of pathogens, including various viruses (e.g., influenza and coronaviruses) that severely threaten the health of humans and/or other animals [4,5,6,7,8]. Leveraging their strategic location, AMs initiate sophisticated defensive mechanisms to clear these invaders [5,8,9,10]. From a developmental perspective, AMs are reported to be differentiated from monocytes, which are myeloid cells that also serve as the sources for various other types of tissue-resident macrophages [2,9,11]. Despite the pivotal functional importance of AMs, the regulators underlying their cell identity and immunoregulatory function remain incompletely understood.

Transcription factors (TFs), a group of proteins that bind cis-regulatory elements (e.g., promoters and enhancers) to control gene expression, are key regulators of embryo development, cell differentiation, as well as cellular response to environment or pathogens [12,13,14]. The cellular functions and response capacities of the immune system are pre-programmed by intrinsic gene regulatory networks, which are known to be dominated by a set of core TFs [15,16,17]. For instance, TCF-1 and GATA3 cooperate to promote innate lymphoid cell differentiation [16,18], RUNX1 and KLF6 modulate neutrophil maturation [19], and FOXP3 is important for regulatory T cells [17]. Moreover, TFs coordinate complex immune processes, including inflammatory response [20], pathogen clearance [21,22], and tissue repair [23]. Notably, some TFs (e.g., PU.1, C/EBPα) also play important roles in monocytes and bone-marrow-derived macrophages [24,25,26,27,28]. Despite these findings, the detailed roles of TFs—including those identified as crucial myeloid regulators—remain largely unexplored in AMs so far.

The transcription factor PU.1, which is encoded by Spi-1 Proto-Oncogene (*SPI1*), is an ETS family protein with critical roles in both myeloid and lymphoid immune cells. In myeloid cells, particularly monocytes and macrophages, PU.1 is indispensable for cell development, lineage specification, and maturation [29,30,31,32,33]. Specifically, PU.1 drives myeloid-specific gene expression by binding to thousands of cis-regulatory elements (CREs), primarily at promoters and enhancers [24,25,27,34]. PU.1 also regulates the development and differentiation of B and T lymphocytes [35,36]. According to the evidence from mouse macrophages and B cells, cell-specific PU.1 binding sites are often enriched for the motifs of multiple collaborative TFs (e.g., CEBPA/B, IRF1), facilitating their synergistic regulation [34,37,38]. Interestingly, recent evidence suggests that a high portion of PU.1-bound enhancer in human monocytes is derived from transposable elements (TEs), which are abundant mobile DNA elements rich in TF-binding motifs and frequently create CREs [25,39,40]. Despite the well-characterized roles of PU.1 in multiple myeloid and lymphoid lineages, the genome-wide binding landscape and regulatory function of PU.1 in AM remain to be characterized.

Pig is an important livestock animal and also a valuable biomedical model [41]. Using porcine AMs (PAMs) as a model, here we combined gene knockdown experiments and multiomic profiling to elucidate the role of PU.1 in AMs. Specifically, we employed Cleavage Under Targets and Tagmentation (CUT&Tag) to characterize the genome-wide occupancy of PU.1 together with the active histone modification H3K27ac, which occupies active promoters and enhancers [16,42,43]. By comparative integration of the epigenomic and RNA sequencing (RNA-seq) data before and after *SPI1* disruption, this study achieves a systematic depiction of the regulatory function of PU.1 in PAMs.

## 2. Materials and Methods

### 2.1. Cell Isolation and Culture

Primary PAMs were collected from two one-month-old healthy piglets via bronchoalveolar lavage (BAL). The piglets were purchased from Taizhou Taihe Biotechnology Co., Ltd. (Taizhou, China), and they had been vaccinated against pseudorabies and Mycoplasma pneumonia with standard protocols. Their health status was confirmed based on the clinical examination of appearance, body weight, respiration, appetite, feces, and urine. Briefly, following tracheal cannulation, the lungs were lavaged with sterile phosphate-buffered saline (PBS). The collected BAL fluid was centrifuged at 600× *g* for 10 min at 4 °C to isolate the cells, which were subsequently washed twice with sterile PBS. For long-term storage, PAMs were resuspended in freezing medium containing 90% fetal bovine serum (FBS; Sigma Aldrich, St. Louis, MO, USA) and 10% dimethyl sulfoxide (DMSO) and then cryopreserved in liquid nitrogen. Revived PAMs were cultured in RPMI-1640 (6125117, Gibco, New York, NY, USA) supplemented with 10% FBS and 1% penicillin-streptomycin (P/S; 15240062, Gibco, New York, NY, USA) and grown in a constant-temperature incubator at 37 °C with 5% CO_2_. The immortalized 3D4/21 cell line was purchased from ATCC and cultured under the conditions as previously described [6].

### 2.2. Gene Knockdown

Small interfering RNA (siRNA) was used to knock down *SPI1* expression. PAMs were cultured in 12-well plates (712001, Nest, Wuxi, China) until they reached 70–80% confluence. Porcine SPI1-specific siRNA was diluted in Opti-MEM (31985062, Gibco, Grand Island, New York, NY, USA) and mixed with transfection reagent (13778150, Invitrogen, Carlsbad, CA, USA) pre-diluted in Opti-MEM. The mixture was incubated in the dark at room temperature for 5 min to form siRNA-liposome complexes, and then added to the cell culture medium for transfection. Samples were collected 24 and 36 h post-transfection. The efficiency of the siRNA experiment was validated by qRT-PCR and Western blot. The sequences of the SPI1 siRNAs are listed in Table S3, and the primer sequences for qRT-PCR are provided in Table S4.

### 2.3. Western Blot

PAMs were lysed with RIPA buffer (P0013B, Beyotime Biotechnology, Nantong, China) supplemented with protease inhibitor cocktail (P1005, Beyotime Biotechnology, Nantong, China) for 20 min on ice, and then centrifuged at 12,000 rpm at 4 °C for 15 min. Protein concentration was determined using the BCA protein assay kit (Solarbio, Beijing, China). Subsequently, equal amounts (20 μg) of denatured proteins were loaded on 10% SDS-PAGE gel for electrophoresis and transferred to PVDF membranes (Immobilon, Darmstadt, Germany). The membranes were blocked with 5% skim milk at room temperature for 1 h and incubated overnight at 4 °C with anti-PU.1 antibody (1:1000, sc-390405, Santa Cruz Biotechnology, Dallas, TX, USA). After washing with 1 × TBST (Solarbio, Beijing, China), the membranes were incubated with HRP-conjugated secondary antibodies (1:5000) at room temperature for 1 h. Protein bands were visualized using the Enhanced Chemiluminescence kit (Abbkine, BMU102, Wuhan, China) and then exposed with Tanon 4600 (Tianneng, Shanghai, China). HSP90 was used as the internal control.

### 2.4. RNA-Seq

Total RNA was isolated using FastPure Cell/Tissue Total RNA Isolation Kit V2 (Vazyme, RC112-01, Nanjing, China). The mRNA was purified from total RNA using poly-T oligo-attached magnetic beads, and then the fragmented mRNA was used for the synthesis of cDNA via reverse transcription. The PCR-amplified cDNA fragments were 300–400 bp long. The libraries were sequenced as 150 bp paired-end reads on the DNBSEQ-T7 platform (BGI, Shenzhen, China).

Raw data were trimmed with Trim Galore! v0.6.4, and then aligned to the pig reference genome (Sscrofa11) using STAR v2.7.3 [44] with default settings. Gene-level read counts were calculated by using the featureCount function of subread v2.0.0 [45]. Differentially expressed genes (DEGs) were identified with DESeq2 v1.30.1 [46] with the cutoff: FDR < 0.05, |log2foldChange| > 1. Transcript per million (TPM) values were calculated with RSEM v1.3.2 [47].

### 2.5. CUT&Tag

CUT&Tag was performed using the NovoNGS CUT&Tag 4.0 High-Sensitivity Kit (Illumina platform, N259-YH01, Novo-protein Scientific Inc., Suzhou, China), following the manufacturer’s protocol. In brief, ~150,000 PAMs were crosslinked with 37% formaldehyde (final concentration 0.1%) at room temperature, and then quenched with glycine. After washing, cells enriched by ConA beads were resuspended in primary antibody buffer containing primary antibody (1:100, sc-390405X, Santa Cruz Biotechnology) and incubated overnight at 4 °C. After discarding the primary antibody buffer, cells were incubated with anti-IgG secondary antibody buffer (1:200) for 1 h. Subsequently, cells were incubated with the proteinA/G-Tn5 transposome complex for 1 h. The tagmentation reaction was then carried out for 1 h. Following termination of the tagmentation reaction, DNA fragments were purified and recovered using magnetic beads, and PCR amplification was performed to construct the sequencing library [43]. The two primary antibodies against PU.1 (1:100, sc-390405X, Santa Cruz Biotechnology) and H3K27ac (1:100, 1044138-15, Abcam, Cambridge, UK) were used in this study.

Raw data were trimmed using Trim Galore! v0.6.4 and aligned to the pig reference genome (Sscrofa11) using Bowtie2 v2.3.5 [48] with the following parameters: --local --very-sensitive-local --no-unal --no-mixed --no-discordant -I 10 -X 1000. PCR duplicates were removed using the rmdup function in SAMtools v1.13 [49]. Following confirmation of high reproducibility between biological replicates, their data were pooled together for further analysis. Peak calling was performed with MACS2 v2.2.6 [50] with settings: --fe-cutoff 2 -q 0.01, -g 2.48e9. Differential binding analysis was performed using DiffBind v3.4.11 [51] with settings: minOverlap = 1, summits = 250, method = DBA_EDGER.

### 2.6. Reference Genome and Annotation

The reference genome and corresponding gene annotation for pig (Sscrofa11) were retrieved from the Ensembl database [52]. TE annotations were obtained from the UCSC Genome Browser [53]. Classification of TE families into evolutionary clades was determined based on the Dfam database [54].

### 2.7. Motif Analysis

The top 1000 peaks with the strongest PU.1 binding signal (ranked by signalValue) were selected for de novo motif enrichment analysis. The analysis was conducted using the findMotifs.pl script from HOMER v4.11 [34], with the parameter: -size 200.

### 2.8. Functional Enrichment Analysis

Gene Ontology (GO) enrichment analysis for gene lists was performed using the DAVID online tool [55] and the R package ChIPseeker v1.40.0 [56]. Gene Set Enrichment Analysis (GSEA) was performed with GSEA v4.3.2 [57].

### 2.9. Epigenetic Annotation of CREs

CREs were classified based on their genomic location and histone modification profiles. Promoters were defined as H3K27ac peaks that are within 500 bp from transcription start sites (TSSs), and enhancers were defined as those that are more than 500 bp away from TSSs.

### 2.10. Genomic Annotation of Adjacent Genes

The genes adjacent to given genomic regions (e.g., PU.1 binding sites or CREs) were determined by comparing with their TSSs using the window function of BEDTools v2.29.2 [58]. The TSS annotation file was retrieved from the Ensembl database using BioMart v2.60.1 [52].

### 2.11. TE Enrichment Analysis

TE enrichment analysis of PU.1 binding sites was performed using the Transposable Element Enrichment Analysis (TEENA) web server [59] with default settings.

### 2.12. Statistical Analysis and Data Visualization

All statistical analyses were performed with the R statistical programming language. Heatmaps for CUT&Tag data were generated using DeepTools v3.5.1 [60]. Heatmaps for RNA-seq data were generated using pheatmap. Representative genomic tracks were visualized using IGV v2.13.1 [61].

## 3. Results

### 3.1. Disruption of PU.1 Impairs the Expression of Many Immune-Related Genes in PAMs

**Figure 1:**
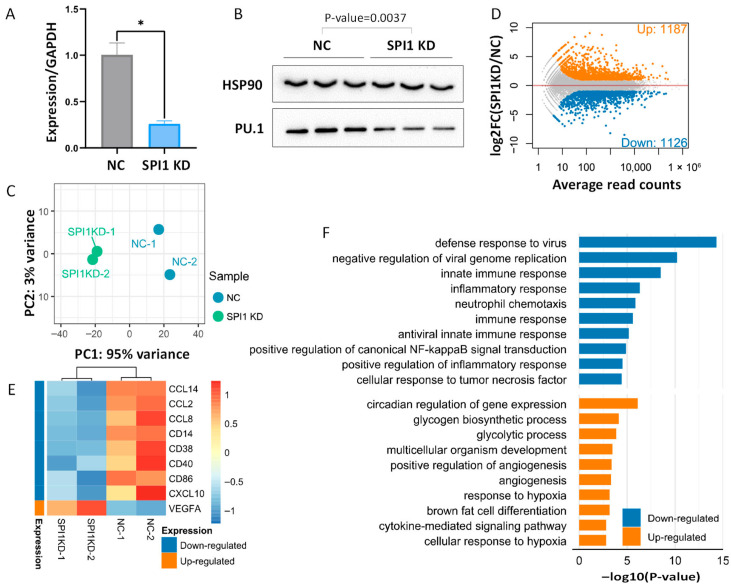
Characterization of the transcriptomic changes after SPI1KD in PAMs. (**A**,**B**) Validation of the *SPI1* knockdown efficiency by using qRT-PCR (**A**) and Western blotting (**B**), respectively. *p*-values are calculated with two-sided Student’s *t*-test. * *p* < 0.05. (**C**) PCA visualization of the RNA-seq replicates for NC and SPI1KD (*n* = 2 per group). NC: negative control; SPI1KD: SPI1 knockdown. (**D**) MA plot for the differential gene expression after SPI1KD based on RNA-seq data. (**E**) Heatmap showing the expression profile of representative DEGs. (**F**) GO enrichment analysis for the DEGs identified between the NC vs. SPI1KD samples.

To determine the regulatory role of PU.1 in PAMs, we performed knockdown experiments to disrupt *SPI1* expression and then applied RNA-seq to characterize the transcriptomic changes. Among the three candidate siRNAs tested for knockdown efficiency, siRNA-840 exhibited the highest silencing efficiency, as demonstrated at both mRNA and protein levels according to qRT-PCR and Western blotting, respectively (Figure 1A,B and Figure S1). Consequently, this siRNA was used for subsequent analysis. We performed RNA-seq on the PAMs transfected with either negative control siRNA (NC) or *SPI1*-targeting siRNA (SPI1KD), with the relevant data summarized in Table S1. Quality control analysis demonstrated high reproducibility among biological replicates, as evidenced by the Principal Component Analysis (PCA) visualization (Figure 1C). We then performed differential expression analysis to identify the genes with affected expression after SPI1KD. A total of 2313 DEGs were identified, including 1126 down-regulated and 1187 up-regulated genes (Figure 1D, Table S2). Therefore, our data suggest that PU.1 exerts remarkable regulatory effects on gene expression in PAMs.

To decipher the biological consequences of SPI1KD, we performed functional enrichment analysis on the identified DEGs. GO analysis revealed that the down-regulated genes are tightly associated with innate immune and inflammatory response (Figure 1E). Consistently, we observed a marked down-regulation of canonical myeloid markers and co-stimulatory molecules, such as *CD14*, *CD40*, and *CD86*, which are pivotal for pathogen recognition and antigen presentation in macrophages [62]. In addition, a broad spectrum of chemokines involved in leukocyte recruitment (e.g., *CXCL10*, *CCL2*, *CCL14*) is also significantly down-regulated (Figure 1F). Then, we further inspected the up-regulated genes and found that they are associated with angiogenesis and developmental pathways (Figure 1F). Among the up-regulated genes, we noticed that *VEGFA* is a growth factor influencing the development and maintenance of the vascular and lymphatic systems and the M1-phenotype of porcine macrophage [63,64]. Accompanying the GO analysis results, we further performed GSEA, which also identified the significant enrichment of gene sets associated with innate immune response (Figure S2). Together, gene knockdown experiments suggest that *SPI1* has an important regulatory function in PAMs, predominantly serving as an activator of innate immune genes.

### 3.2. PU.1 Frequently Binds to Epigenetically Annotated Promoters and Enhancers

After uncovering the PU.1-mediated regulation on immune-related genes in PAMs, we further applied CUT&Tag to delineate the genome-wide binding landscape of PU.1 together with the histone modification H3K27ac, which occurs primarily at active promoters and enhancers [42]. To achieve better characterization of the chromatin state of PU.1 binding loci, we also collected the matched public epigenomic data for integrative analysis. These data include: (1) Assay for Transposase-Accessible Chromatin with high-throughput sequencing (ATAC-seq) data, which measures chromatin accessibility; (2) CUT&Tag data for the histone modification H3K4me3, which mainly occurs at active promoters; (3) CUT&Tag data for the histone modification H3K27me3, which mainly occurs at heterochromatin. The details for all these data are summarized in Table S1.

**Figure 2:**
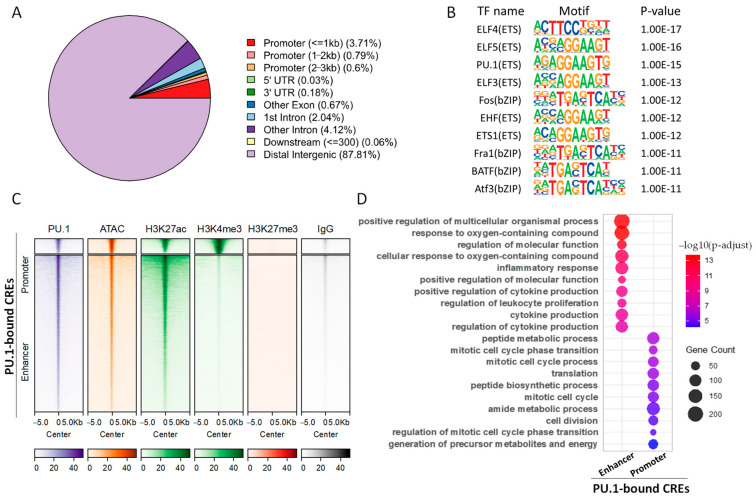
Characterization of the genome-wide binding and epigenomic profiles of PU.1 in PAMs. (**A**) Genomic distribution of PU.1 binding sites. (**B**) Top enriched TF motifs in PU.1 binding sites. (**C**) Epigenetic patterns for the promoter-proximal and distal PU.1 binding sites. (**D**) GO enrichment results for the promoter-proximal and distal PU.1 binding sites.

A total of 69,794 PU.1 peaks were identified, with 5.1% occurring in promoters, while the majority (87.8%) of the remaining occur in distal intergenic regions (Figure 2A). As expected, the canonical ETS-family motif is significantly enriched in PU.1 binding loci (Figure 2B). PU.1 binding loci are usually highly accessible and enriched with active histone marks (i.e., H3K27ac and H3K4me3) yet depleted of the repressive histone mark H3K27me3 (Figure 2C and Figure S3), indicating that it predominantly binds active CREs. We further classified PU.1 binding sites into promoter-proximal and distal groups, and confirmed their distinct chromatin signatures as illustrated by the strong H3K27ac signal shared by both groups and remarkably higher H3K4me3 signal for promoter regions (Figure 2C). Notably, GO enrichment analysis indicates that PU.1-bound promoters are primarily enriched in fundamental metabolic processes related to the basal functions and proliferative capacity of macrophages, while PU.1-bound enhancers are specifically enriched for the pathways related to immune cell activation, effector function execution, and homeostasis maintenance (Figure 2D). We further performed KEGG enrichment analysis and observed similar patterns (Figure S4). Collectively, PU.1 mainly binds to active promoters and enhancers in PAMs, with the bound enhancers tightly associated with genes with important immune functions.

### 3.3. Distinct TE Sub-Families Contribute High Proportions of PU.1-Bound Enhancers

**Figure 3:**
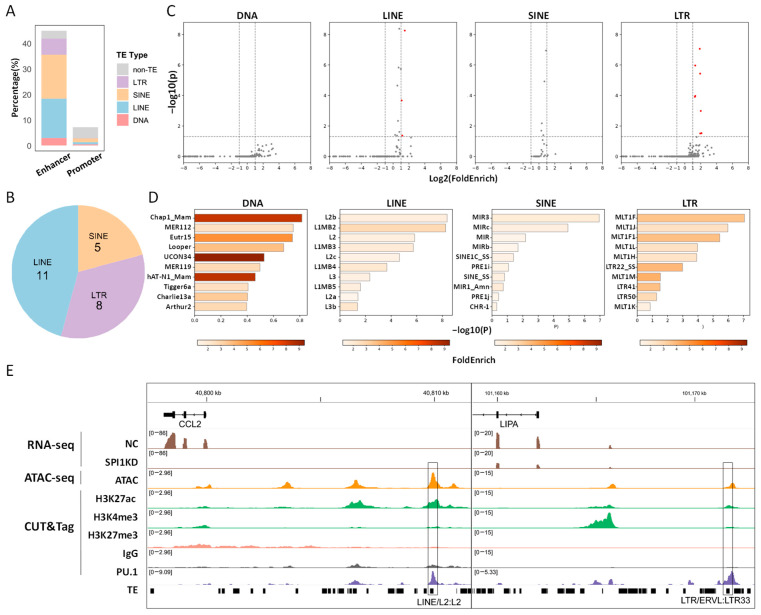
Characterization of the association between TEs and PU.1-bound CREs in PAMs. (**A**) The proportions of PU.1-bound enhancers and promoters that overlap major TE classes, including LTR, LINE, SINE, and DNA transposons. (**B**) Pie chart showing the proportions of significantly enriched TE sub-families within PU.1-bound enhancer that belong to each TE class. (**C**) Volcano plots showing the global TE enrichment patterns for PU.1-bound enhancer peaks. Significantly enriched TE sub-families are highlighted in red color. (**D**) Bar plots showing the enrichment of distinct TE sub-families within PU.1-bound enhancers. The top 10 TE sub-families, as ranked by *p*-value, are presented. (**E**) IGV tracks show representative TE-derived enhancers that likely mediate PU.1-dependent regulation of immune-related genes. The boxes highlight the positions of TE-derived enhancers.

Recent studies suggest that TEs frequently create CREs (particularly enhancers) in human and/or mouse T/B lymphocytes and monocytes [25,65,66,67]. Intrigued by these findings, we analyzed the enrichment of TEs within PU.1-bound CREs in PAMs. Overall, a much higher TE overlapping ratio is observed in PU.1-bound enhancers relative to promoters (Figure 3A), which is as expected given the similar trend reported in other cell types in multiple previous studies [13,25,68,69]. We identified *24* significantly enriched TE sub-families in PU.1-bound enhancers, most of them belonging to long interspersed nuclear element (LINE), short interspersed nuclear element (SINE), or long-terminal repeat (LTR) retrotransposons (Figure 3B,C, Table S3). In contrast, no TE sub-family is enriched in PU.1-bound promoters (Figure S5), so we focused the analysis only on PU.1-bound enhancers. Interestingly, apart from ancient TE sub-families (e.g., L2b and MLT1J, which are shared by theria), multiple enriched sub-families (e.g., LTR22_SS and SINE1C_SS) are pig-specific (Figure 3D, Table S3), therefore have the potential to facilitate the formation of pig-specific enhancers. We further demonstrate that the TE-derived enhancers bound by PU.1 are associated with myeloid and immune-inflammatory functions, exhibiting a pronounced immune regulatory signature (Figure 3E). Manual inspection identifies many immune-related genes adjacent to TE-derived enhancers. For instance, an L2-derived enhancer lies ~9.9 kb upstream of *CCL2* (Figure 3E), which is a central chemokine governing monocyte/macrophage recruitment to inflammatory sites. In addition, an LTR33-derived enhancer is ~9.6 kb upstream of *LIPA* (Figure 3E), which is an enzyme regulating macrophage lipid homeostasis and lysosome-dependent inflammatory activation. Collectively, distinct TE sub-families contribute to the formation of PU.1-bound enhancers in PAMs, including many that may have the potential to regulate adjacent immune-related genes.

### 3.4. Epigenomic Identification of the Putative CREs Bound and Regulated by PU.1

**Figure 4:**
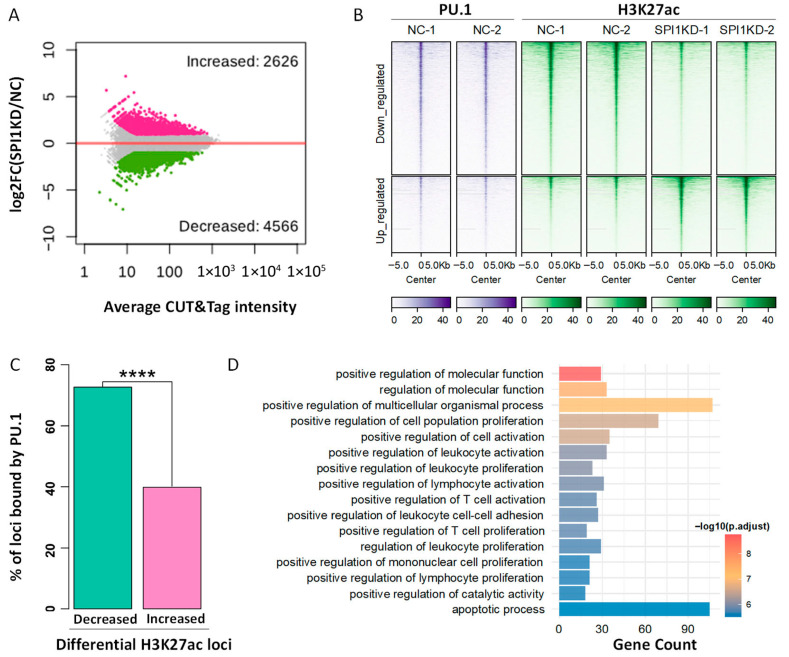
Characterization of the PU.1-regulated CREs in PAMs. (**A**) MA plot showing the differential H3K27ac intensity after SPI1KD based on CUT&Tag data. The loci with significantly increased and decreased H3K27ac levels are highlighted in red and green, respectively. (**B**) Heatmaps showing epigenetic characteristics of PU.1-bound CREs that show reduced H3K27ac levels after SPI1KD. (**C**) Comparison of up-regulated (Increased) vs. down-regulated (Decreased) H3K27ac loci regarding their overlap with PU.1 peaks. The *p*-value calculated by using one-sided Fisher’s Exact Test is indicated. **** *p* < 0.0001. (**D**) GO enrichment results for the down-regulated H3K27ac loci, which are bound by PU.1.

To further identify the CREs under the regulation of PU.1 in PAMs, we applied CUT&Tag to determine the changes in the active histone mark H3K27ac after SPI1KD. In total, we identified 2626 and 4566 genomic loci with increased and decreased H3K27ac level, respectively (Figure 4A,B). Notably, 77.2% (n = 3524) of the H3K27ac-decreased loci are bound by PU.1, which is of a much higher proportion than the 43.8% (n = 1150) for H3K27ac-increased loci (Figure 4C). These data indicate that PU.1 primarily acts as a transcriptional activator in PAMs, consistent with previous observations in human and mouse monocytes [25,70]. Notably, a large amount of TE-derived CREs bound by PU.1 also show altered H3K27ac level after SPI1KD (Figure S6). We further determined the functional relevance of the 3524 PU.1-bound loci showing decreased H3K27ac level after SPI1KD, which are considered as the putative CREs under direct regulation of PU.1. GO enrichment analysis demonstrates that these PU.1-regulated loci are associated with multiple immune-related biological processes, such as regulation of leukocyte activation, cytokine production, and inflammatory responses (Figure 4D). Similarly, KEGG analysis also reveals their association with immune and pathogen-related pathways, such as influenza A, measles, leishmaniasis, pertussis, and cytokine-receptor interaction (Figure S7). Together, epigenomic comparison uncovers thousands of CREs likely under the direct regulation of PU.1, and these CREs are highly associated with immune-related genes.

### 3.5. Integrative Analysis Indicates the Direct Regulation of Crucial Immune Genes by PU.1

**Figure 5:**
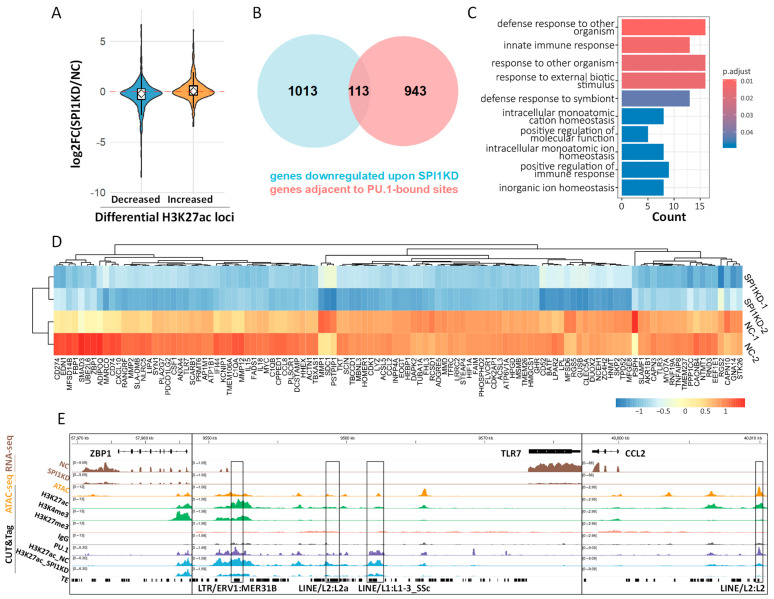
Identification of the putative genes under direct regulation of PU.1 in PAMs. (**A**) Violin plot showing the global transcriptional changes in the genes flanking H3K27ac-increased and H3K27ac-decreased loci, respectively. (**B**) Venn diagram illustrating the identification of direct target genes of PU.1, defined as the intersection between genes downregulated upon SPI1KD and genes adjacent to PU.1-regulated CREs. (**C**) GO enrichment result for the putative genes under direct regulation of PU.1. (**D**) Expression profile of the putative immune genes with altered expression after SPI1KD. (**E**) IGV tracks showing representative immune genes likely under the direct regulation of PU.1, including *ZBP1*, *TLR7*, and *CCL2*.

Our genomic profiling revealed that PU.1 occupies over 70,000 genomic sites in PAMs, predominantly localized to promoters and enhancers. While SPI1KD led to broad transcriptomic changes, further inference of direct causality requires simultaneous consideration of PU.1 binding with epigenetic and transcriptional responses. First, we examined the expression changes for the genes associated with H3K27ac-decreased loci. As expected, the genes adjacent to H3K27ac-decreased loci show a global trend of decreased expression after SPI1KD, which is in contrast to H3K27ac-increased loci (Figure 5A). Then, we computationally screened for the putative direct PU.1-regulated genes, defined as PU.1-activated genes (i.e., genes showing decreased expression after SPI1KD) that are adjacent to PU.1-activated loci (i.e., PU.1-bound loci showing decreased H3K27ac level after SPI1KD). In total, we identified 113 genes as putative direct targets of PU.1 (Figure 5B). Interestingly, GO enrichment analysis suggests that those genes are highly associated with immune-related functions, such as response to other organisms, and innate immune response (Figure 5C). Importantly, dozens of them are annotated as immune-related genes according to the GO results (Figure 5D,E). For example, ZBP1 is a critical innate immune-related protein capable of sensing Z-form DNA and RNA molecules [71,72], *TLR7* is a Toll-like receptor that recognizes viral RNA [73], and *CCL2* is a chemokine governing monocyte/macrophage recruitment [74]. The down-regulation of *ZBP1* and *TLR7* is further validated by qRT-PCR (Figure S8). Notably, *TLR7* and *CCL2* are adjacent to a putative PU.1-activated enhancer derived from LTR or LINE retrotransposons (Figure 3E and Figure 5E), further highlighting the contribution of TEs to the PU.1-mediated cis-regulatory network. Together, computational screening identifies many immune-related genes likely under the direct regulation of PU.1, supporting the important immunoregulatory function of PU.1 in PAMs.

## 4. Discussion

Alveolar macrophages serve as the first line of defense in the respiratory tract, playing essential roles in maintaining lung homeostasis and orchestrating immune responses against inhaled pathogens [2,9]. Despite their critical functions, the transcriptional regulatory networks governing AMs remain poorly understood compared to other immune cell populations, such as monocytes and B/T lymphocytes. Furthermore, while many TFs are known to be important instructors for cell identity and function [3,15,16], the precise regulatory landscape of the core myeloid TF PU.1 within the unique microenvironment of the alveolar niche has not been systematically investigated. To address this knowledge gap, our study integrated multiomic profiling and molecular experiments to systematically elucidate the function of PU.1 in PAMs.

Our first major finding is about the genome-wide binding characteristics of PU.1 in PAMs. We demonstrate that PU.1 predominantly binds to active CREs and is associated with the regulation of adjacent genes enriched in immune-related functions. This observation is consistent with the well-established role of PU.1 as a master regulator of myeloid gene expression via promoter and enhancer occupancy [27,34]. Although PU.1 has not been extensively characterized in either AMs or any porcine cells, our findings align with reports in human and murine monocytes and macrophages, where PU.1 similarly binds active CREs and regulates adjacent immune-related genes [25]. This similarity likely reflects the developmental origin of AMs from monocytes [2,9], supporting a core transcriptional program for macrophage identity. Therefore, our data fills an important gap in porcine immunology and underscores the functional conservation of PU.1 across myeloid lineages and mammalian species. Further comparative studies in human and mouse AMs will help to clarify the cross-species conservation of PU.1-dependent regulatory network across mammalian species.

A second notable finding is the frequent association between PU.1-bound enhancers and TEs, which are widespread mobile DNA elements in mammalian genomes. While TE-derived enhancers have been extensively studied in humans and mice, the cis-regulatory role of porcine TEs remains largely unexplored. In human and mouse, TE-derived CREs contribute to various biological processes, including embryonic development, aging, and tumorigenesis [68,75,76,77,78], and their cis-regulatory contribution in the immune system has also been reported [25,65,67,79,80,81,82]. In contrast, studies on the cis-regulatory roles of porcine TEs have just begun to appear in recent years. For example, we previously uncovered the links between TEs and 3D chromatin structure in porcine spleen [83], and other researchers reported the potential cis-regulatory roles of porcine TEs in muscle and other tissues [84,85,86]. In PAMs, we found that PU.1 can bind both ancient TE (e.g., L2) and pig-specific sub-families (e.g., LTR22_SS, SINE1C_ss). Importantly, many genes adjacent to TE-derived enhancers have important immune functions, such as *TLR7*, which is a Toll-like receptor for viral RNA recognition [73], and *CCL2*, which is a chemokine for monocyte/macrophage recruitment [74]. This suggests that many PU.1-regulated TEs may form enhancers to drive the expression of immune-regulated genes. Given that multiple identified TE sub-families are pig-specific, we speculate that they may have the potential to drive pig-specific expression of some immune-related genes and facilitate the immune response against the spectrum of porcine-specific pathogens, such as PRRSV, ASFV, and PEDV. So far, the cis-regulatory roles of TEs in immune evolution have only been reported in humans, mice, and a few other animals [80,81], yet remain elusive in pigs so far. While further functional validation is beyond the scope of our work, we expect that future studies employing CRISPR-mediated perturbation could validate the functions of representative TE-derived enhancers in infection resistance and immune defense in PAMs.

Finally, the other contribution of this study is the functional characterization of PU.1 in PAMs, achieved through SPI1KD coupled with comparative multiomic analysis. Although PU.1 is well-studied in general monocyte-macrophage biology, where it is important for cell maturation and lineage specification [31,33], its specific regulatory role within the alveolar microenvironment has been largely overlooked. Our results demonstrate that PU.1 is important for the regulation of a broad spectrum of immune-related genes. Mechanistically, we elucidated how PU.1 modulates these genes by directly binding to their associated CREs. It is important to note that even in human and murine models, dedicated studies focusing specifically on the functions of TFs (such as PU.1) in AMs are rare. Therefore, our findings represent a substantial contribution to the AM research field. From an evolutionary perspective, this work establishes a foundation for future parallel studies in humans or mice. Given the unique immune challenges faced by the lung, comparative analyses of AMs across species may reveal fascinating evolutionary adaptations in transcriptional regulation.

## 5. Conclusions

In summary, this study integrated multiomic profiling and gene knockdown experiments to characterize the regulatory role of PU.1 in PAMs at the genome-wide level. Our data reveal the binding and regulatory properties of PU.1 and highlight its important roles in regulating many immune-related genes, including those crucial for immune capacity and pathogen response of AMs. Overall, this study provides novel insights into the regulatory landscape and functional importance of PU.1 in PAMs and extends our knowledge about this core regulator in the mammalian immune system.

## Data Availability

All the data generated in this study have been deposited in the NCBI Gene Expression Omnibus (GEO) under accession GSE318787. This study also re-analyzed public data from previous study [87], which is retrived from GEO with accession GSE231435. All these data are summarized in Table S1.

## Supplementary Materials

The following supporting information can be downloaded at https://www.mdpi.com/article/10.3390/ani16071116/s1, Figure S1: Validation of the knockdown efficiency of the three synthesized SPI1 siRNAs; Figure S2: GSEA results based on the RNA-seq data of the NC and SPI1KD groups of PAMs; Figure S3: Epigenetic patterns of PU.1 binding sites in PAMs; Figure S4: KEGG enrichment results of PU.1-bound CREs in PAMs; Figure S5: TE enrichment results of PU.1-bound promoters in PAMs; Figure S6: Alteration of H3K27ac level of TE-derived CREs bound by PU.1 after SPI1KD; Figure S7: KEGG enrichment result for the putative PU.1-regulated CREs in PAMs; Figure S8: qRT-PCR validation of representative DEGs upon SPI1 knockdown; Table S1: Sources of data used in this study (GSE231435); Table S2: List of DEGs in PAMs following SPI1KD; Table S3: Sequences of siRNAs used for *SPI1* knockdown; Table S4: Primer sequences used for qRT-PCR experiments.
