# Supplementary material for: Multiomic Profiling Reveals the Regulation of Many Immune-Related Genes by PU.1 in Porcine Alveolar Macrophages
Source: Animals (Basel). 2026 Apr 5;16(7):1116. doi: 10.3390/ani16071116 (PMC13072208; doi:10.3390/ani16071116)
Supplement: Supplementary file 1 [file animals-16-01116-s001.zip › Supplemental Figures.pdf]

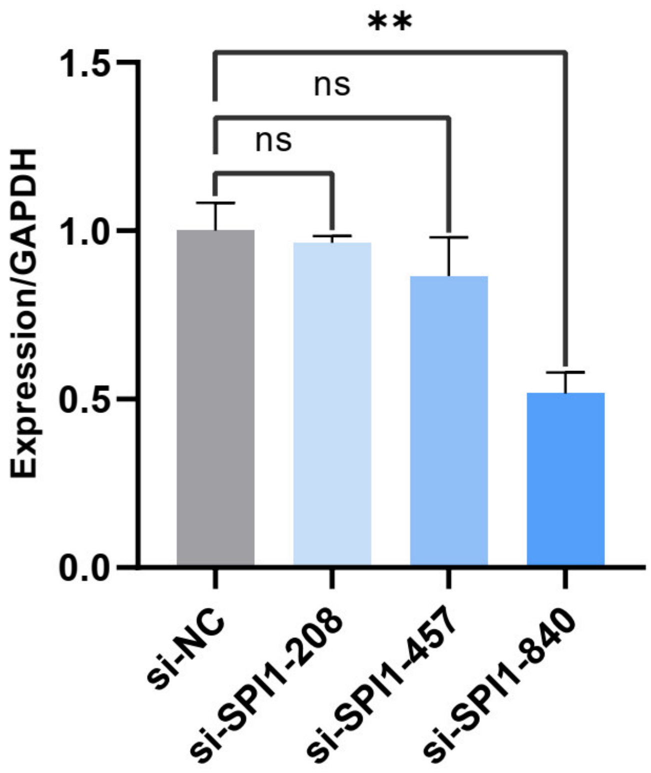

Figure S1 Validation of the knockdown efficiency of the three synthesized SPI1 siRNAs
The qRT-PCR validation of SPI1 knockdown efficiency in 3D4/21 cells showed that si-SPI1-840 exhibited the strongest knockdown effect.

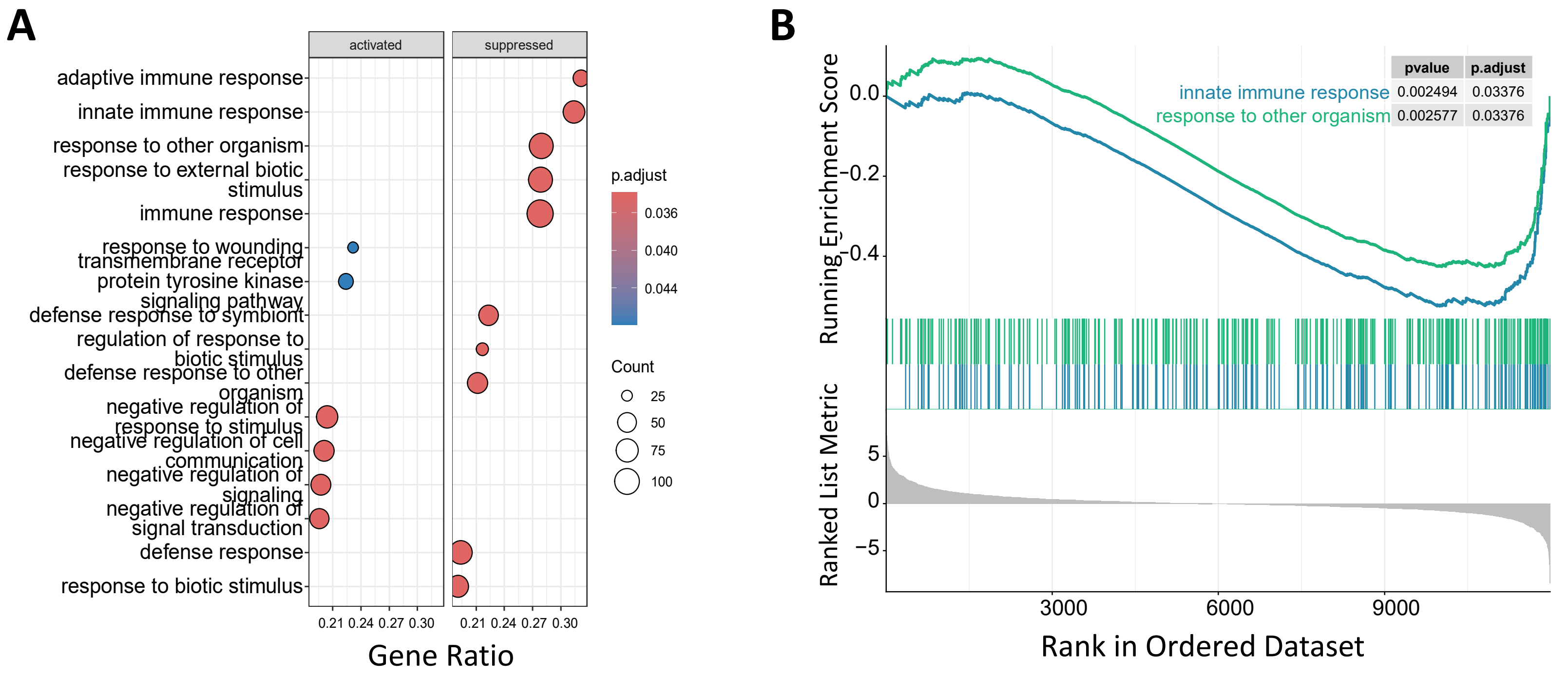

Figure S2 GSEA results based on the RNA-seq data of the NC and SPI1KD groups of PAMs

(A) Dot plots showing the top enriched terms from GSEA analysis. (B) Visualization of the enrichment pattern for the two representative immune-related gene sets.

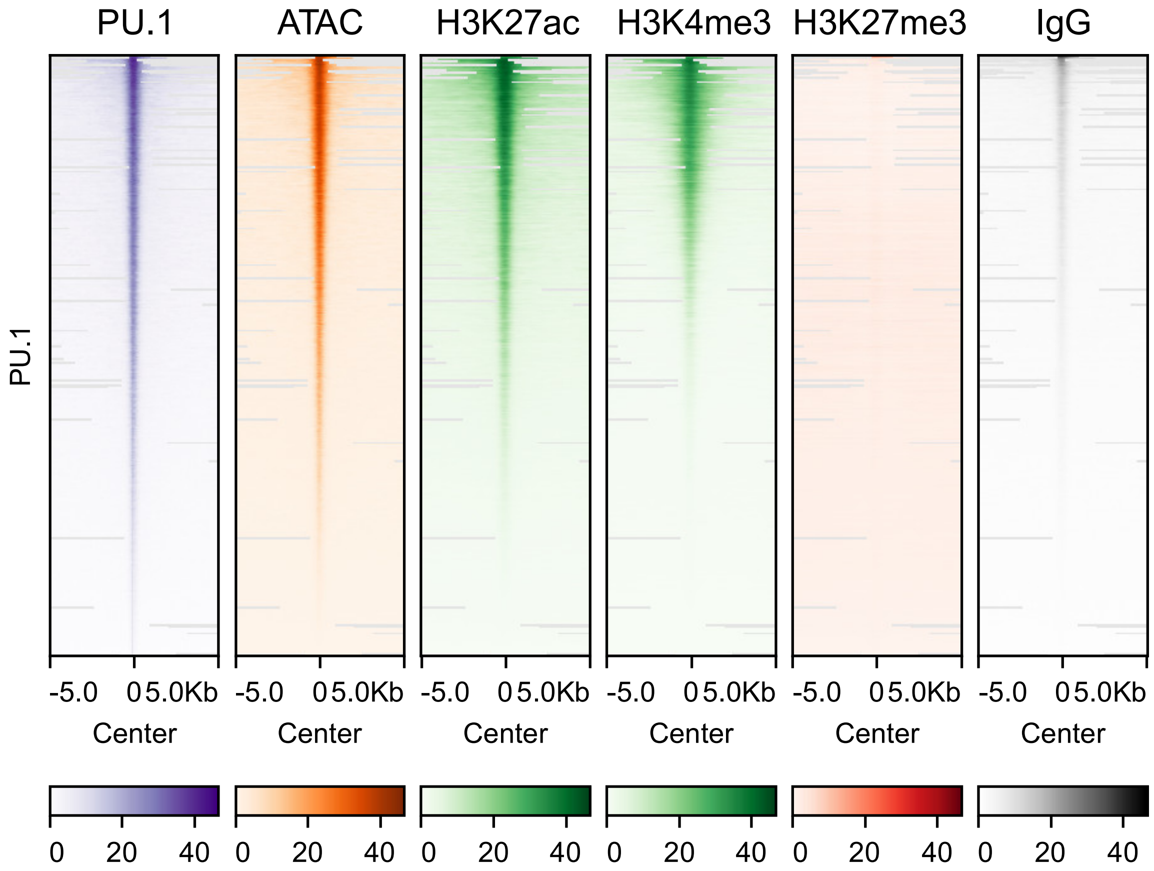

Figure S3 Epigenetic patterns of PU.1 binding sites in PAMs

The heatmap visualizes the binding profiles of PU.1 together with different histone modifications at PU.1-binding sites in PAMs.

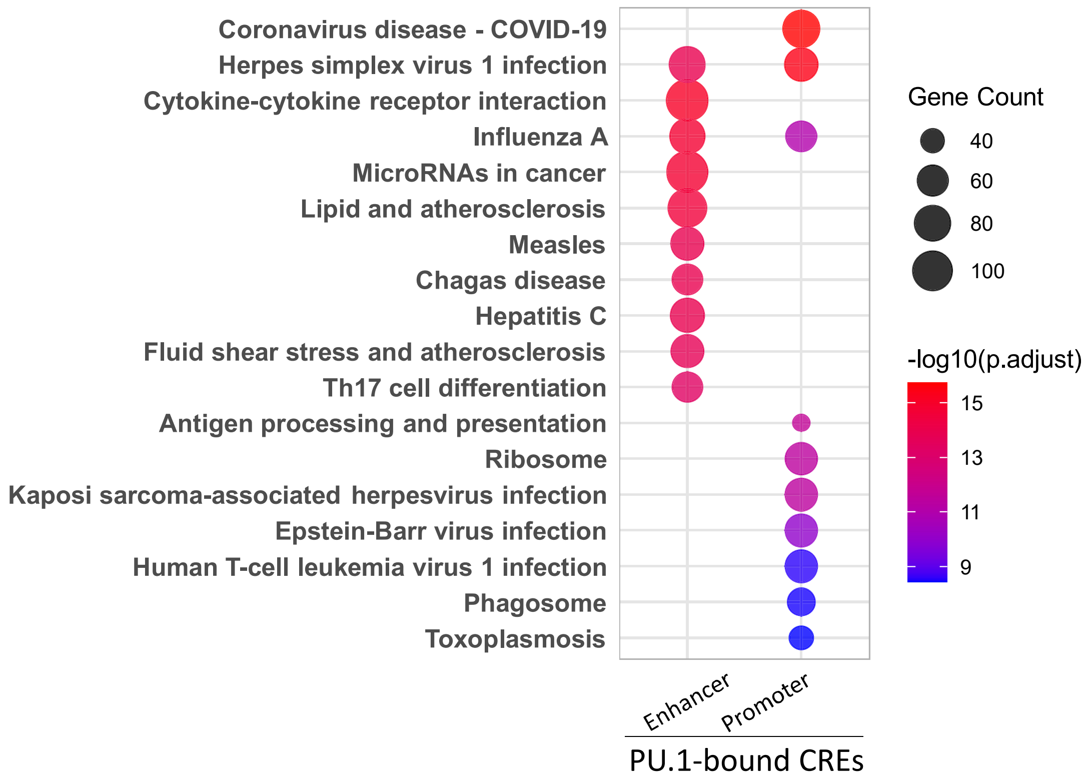

Figure S4 KEGG enrichment results of PU.1-bound CREs in PAMs

KEGG enrichment results for the promoter-proximal and distal CREs bound by PU.1 in PAMs

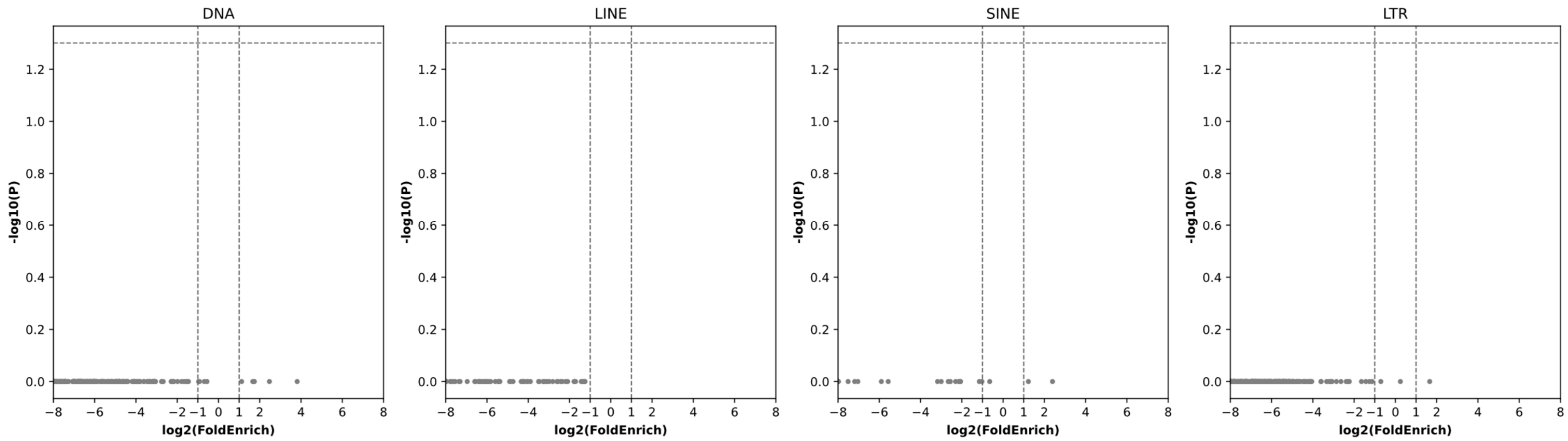

Figure S5 TE enrichment results of PU.1-bound promoters in PAMs

The TE enrichment profiles are visualized as volcano plots. This figure is generated for PU.1-bound promoters by using the TEENA webserver with default settings.

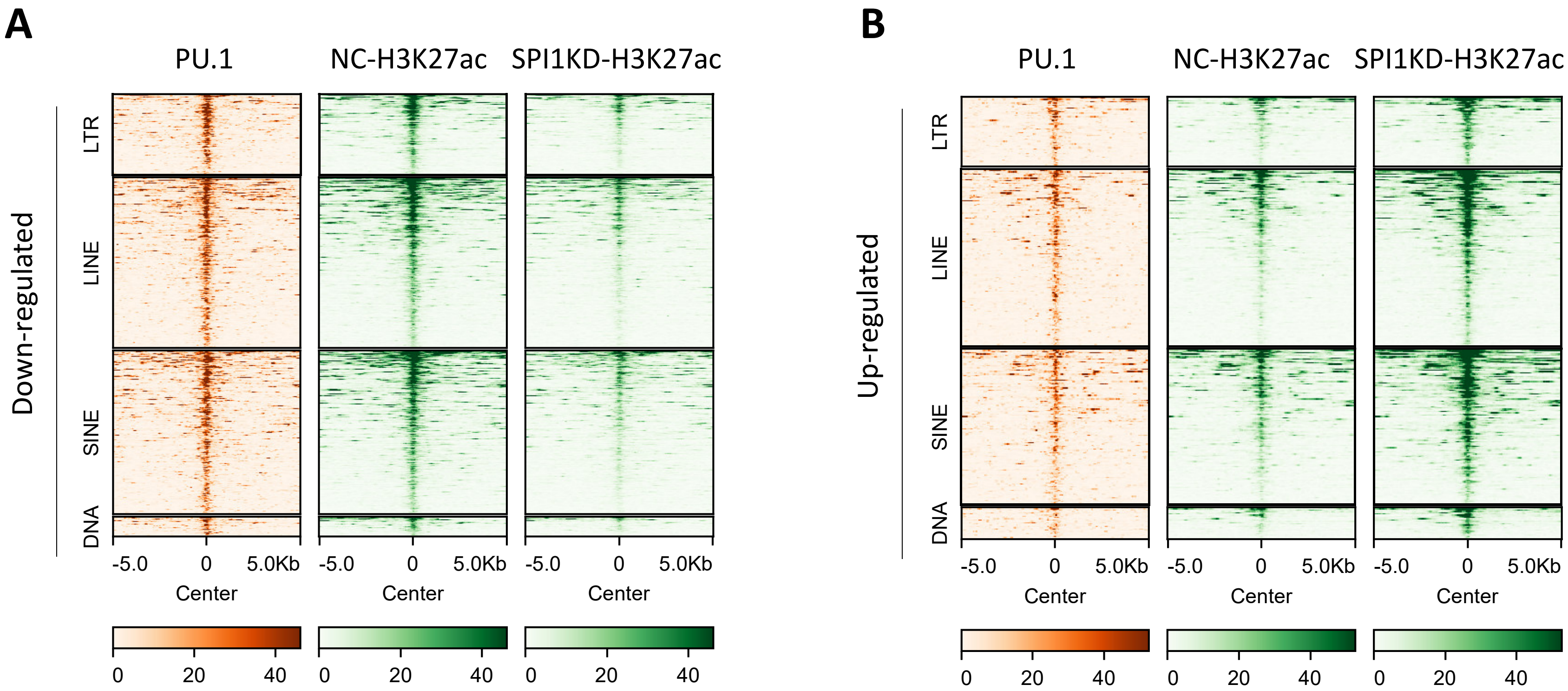

Figure S6 Alteration of H3K27ac level of TE-derived CREs bound by PU.1 after SPI1KD

The heatmaps show the alteration of H3K27ac intensity at PU.1-bound TE-derived CREs after SPI1KD in PAMs. The PU.1-bound CREs with decreased (A) or increased (B) levels of H3K27ac after SPI1KD are visualized separately.

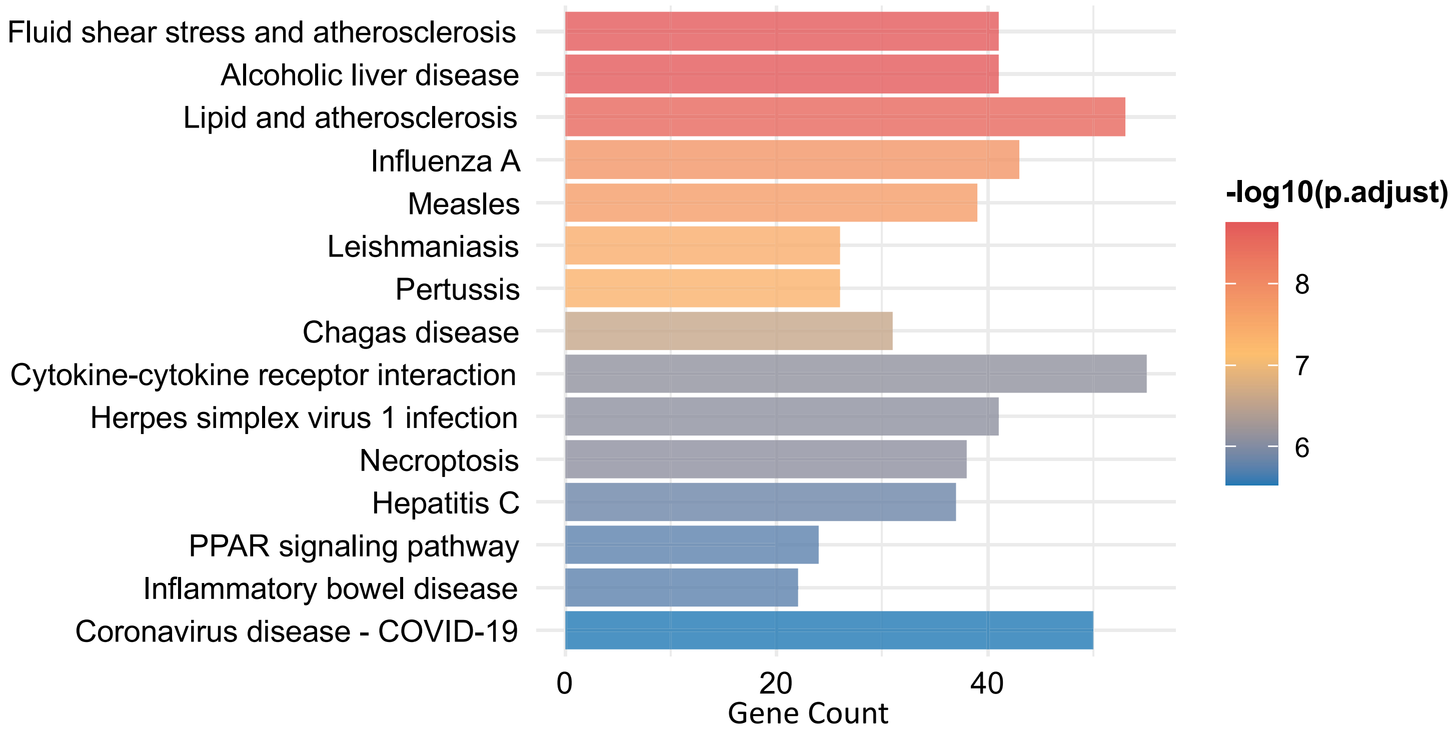

Figure S7 KEGG enrichment result for the putative PU.1-regulated CREs in PAMs

KEGG enrichment results for the PU.1-bound CREs showing decreased H3K27ac level after SPI1KD. The top 15 most significant GO terms are included for visualization.

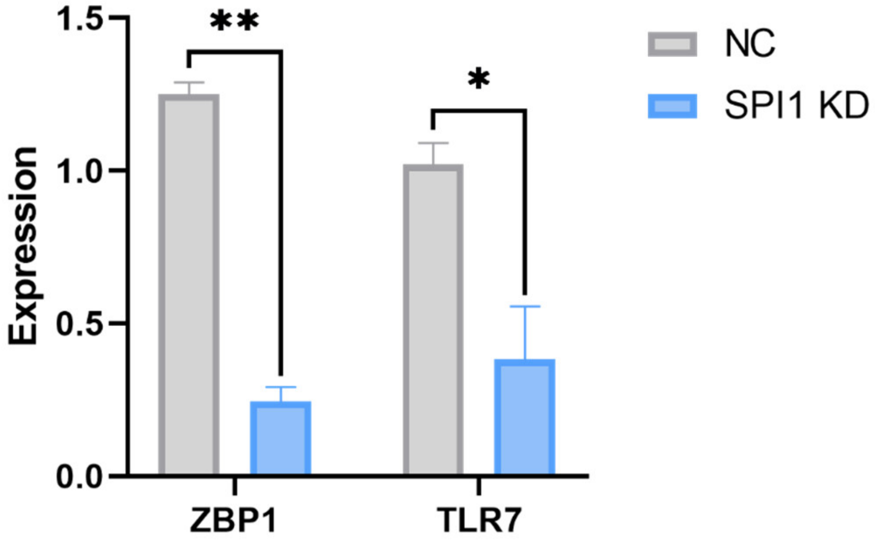

Figure S8 qRT-PCR validation of representative DEGs upon SPI1 knockdown.
The qRT-PCR experiment confirmed the reduced expression of the two representative DEGs including ZBP1 and TLR7, which are identified by RNA-seq data..
