# Supplementary material for: Multiomic Profiling Reveals the Regulation of Many Immune-Related Genes by PU.1 in Porcine Alveolar Macrophages
Source: Animals (Basel). 2026 Apr 5;16(7):1116. doi: 10.3390/ani16071116 (PMC13072208; doi:10.3390/ani16071116)
Supplement: Supplementary file 1 [file animals-16-01116-s001.zip › Table S3.pdf]

Table S3. Sequences of siRNAs used for *SPI1* knockdown

**Name**	**Sense sequence (5'→3')**	**Antisense sequence (5'→3')**
Pig-siSPI1-208	GGAUCUCUACCAACGCCAATT	UUGGCGUUGGUAGAGAUCCTT
Pig-siSPI1-457	CCACCAGGUUUCCUACCUATT	UAGGUAGGAAACCUGGUGGTT
Pig-siSPI1-840	GCAAGACGGGCGAGGUCAATT	UUGACCUCGCCCGUCUUGCTT
Negative Control FAM	UUCUCCGAACGUGUCACGUTT	ACGUGACACGUUCGGAGAATT
