# Supplementary material for: Multiomic Profiling Reveals the Regulation of Many Immune-Related Genes by PU.1 in Porcine Alveolar Macrophages
Source: Animals (Basel). 2026 Apr 5;16(7):1116. doi: 10.3390/ani16071116 (PMC13072208; doi:10.3390/ani16071116)
Supplement: Supplementary file 1 [file animals-16-01116-s001.zip › Table S4.pdf]

Table S4. Primer sequences used for qRT-PCR experiments

Gene name	Primer sequence (5'→3')	Sequence length (bp)
Pig-*GAPDH*	F: ACATCATCCCTGCTTCTACTGG<br>R: CTCGGACGCCTGCTTCAC	188
Pig-*SPI1*	F: GGCAACCGCAAGAAGATGAC<br>R: CCGCTGAACTGGTAGGTGAG	110
Pig-*ZBP1*	F:ATGGCAACGAGATGAGACT<br>R:AGGAAGCACGAGCGAATT	184
Pig-*TLR7*	F:GAGTGGGTTTTGGATGAGCT<br>R:CTCTGGGAAAGGTTTTCCAG	122
